# The effect of progressive muscle relaxation exercises on primary dysmenorrhea, menstrual symptoms, and quality of life: a randomized controlled trial

**DOI:** 10.1007/s11845-026-04410-2

**Published:** 2026-05-01

**Authors:** Sıdıka Pelit Aksu, Aliye Doğan Gangal, Mehtap Uzun Aksoy, Ayten Şenturk Erenel

**Affiliations:** 1 Faculty of Nursing, Department of Obstetrics and Gynecology Nursing Ankara, Gazi University, Ankara, Turkey; 2 Faculty of Nursing, Department of Obstetrics and Gynecology Nursing Konya, Selcuk University, Konya, Turkey

**Keywords:** Primary dysmenorrhea, Menstrual symptoms, Progressive muscle relaxation, Quality of life

## Abstract

**Background:**

Primary dysmenorrhea is a common gynecological condition that negatively affects daily functioning and quality of life in young women. Non-pharmacological interventions such as progressive muscle relaxation exercises (PMRE) may provide supportive benefits in symptom management.

**Aim:**

This study aimed to evaluate the effects of PMRE on pain intensity, menstrual symptoms, and quality of life in young women with primary dysmenorrhea.

**Methods:**

This single-center, parallel-group randomized controlled trial included 42 young women with primary dysmenorrhea who were randomly assigned to intervention and control groups. Pain intensity, menstrual symptom intensity, and quality of life were assessed across two menstrual cycles. The trial was registered at ClinicalTrials.gov (NCT05340101).

**Results:**

Menstrual symptom intensity and pain scores were significantly lower in the intervention group compared with the control group (*p* < .001). A significant between-group difference was observed in the mental component of quality of life in favor of the intervention group (*p* = .001), whereas no significant difference was found in the physical component (*p* > .05). Repeated-measures analyses also demonstrated significant time effects and group × time interactions, indicating a reduction in dysmenorrhea intensity over time in the intervention group (*p* < .05).

**Conclusion:**

PMRE was associated with reductions in dysmenorrhea intensity and menstrual symptoms, as well as improvements in the mental component of quality of life. These findings suggest that structured relaxation exercises may serve as a feasible complementary approach for symptom management in primary dysmenorrhea.

## Introduction

Dysmenorrhea is characterized by pelvic pain and cramping in the lower abdomen occurring during menstruation, often accompanied by nausea, dizziness, fatigue, and headache [1, 2]. Although the pathogenesis of primary dysmenorrhea remains uncertain, current evidence suggests a correlation between increased prostaglandin secretion and myometrial contractions [3]. According to recent systematic reviews and meta-analyses, the prevalence rates of primary dysmenorrhea in studies vary widely, ranging from 66.1% to 71.1% [4, 5].

Primary dysmenorrhea is not limited to pain. It is often accompanied by various menstrual symptoms that can affect daily life and emotional well-being. These symptoms include fatigue, headache, nausea, breast tenderness, irritability, anxiety, sleep problems, and difficulty concentrating. These symptoms can occur together with menstrual pain and increase the overall burden experienced by women. As a result, they may negatively affect daily functioning and how women perceive their health [6]. While women with mild dysmenorrhea may report short-term symptoms with minor impacts on quality of life, those experiencing moderate to severe dysmenorrhea face notable reductions in quality of life [7]. Dysmenorrhea and related menstrual symptoms can negatively affect quality of life. Quality of life includes physical health, emotional well-being, social relationships, and daily functioning. Repeated menstrual pain and accompanying symptoms may cause absence from school or work, reduced participation in daily and social activities, emotional distress, and lower life satisfaction. Therefore, evaluating quality of life helps to better understand the overall impact of primary dysmenorrhea, beyond pain alone [5, 8]. Moreover, dysmenorrhea is associated with heightened levels of depression, anxiety, and negative self-perception [9].

The standard management options for primary dysmenorrhea include nonsteroidal antiinflammatory drugs (NSAIDs) and hormonal contraceptives. However, NSAIDs do not consistently provide complete pain relief and may cause adverse gastrointestinal and neurological effects. Similarly, hormonal contraceptives may be unsuitable for some patients because of contraindications to comorbid medical conditions or because of their potential side effects [10]. These limitations highlight the need for reliable and easy-to-apply non-pharmacological management strategies for primary dysmenorrhea [11].

Given the multidimensional nature of primary dysmenorrhea, effective interventions should aim not only to reduce pain but also to alleviate menstrual symptoms and improve overall quality of life. Progressive muscle relaxation exercises (PMRE), as a safe and easily applicable non-pharmacological intervention, have been shown to promote psychological and physiological relaxation responses and support autonomic regulation, which may contribute to improved overall well-being [12]. PMRE, a mind–body therapy, has shown promise in managing pain and alleviating psychological distress [13]. Following guided instructions, progressive muscle relaxation involves the sequential contraction and relaxation of specific muscle groups. This technique, based on the conscious control of repeated muscle contractions and relaxations, reduces sympathetic nervous system activity and promotes overall bodily relaxation [14]. Evidence suggests that PMRE is effective in reducing perceived pain intensity and improving psychological well-being across various patient populations [15, 16]. However, despite these potential benefits, the independent effectiveness of PMRE in reducing pain, alleviating menstrual symptoms, and improving quality of life among women with primary dysmenorrhea remains insufficiently investigated.

Some studies indicate that PMRE can effectively alleviate pain intensity in primary dysmenorrhea; nonetheless, these studies were conducted alongside other interventions, such as hot compression and aerobic exercise, without isolating the effects of PMRE [17–19]. Few randomized controlled trials have examined the effect of PMRE on primary dysmenorrhea [20, 21]. Further empirical evidence is needed to clarify the effectiveness of PMRE for pain relief, menstrual symptoms, and quality of life among women with primary dysmenorrhea. We hypothesized that PMRE is a practical and reliable approach for managing primary dysmenorrhea and enhancing young women’s quality of life. This study aimed to evaluate the effects of PMRE on pain intensity, menstrual symptoms, and quality of life in young women with primary dysmenorrhea.

## Methods

### Study design

This study was a single-center, parallel-group randomized controlled trial with a pretest–posttest design. The trial was registered on ClinicalTrials.gov (NCT05340101) and conducted in accordance with the Consolidated Standards of Reporting Trials (CONSORT) guidelines.

### Population and sample

The study population consisted of third- and fourth-year young women enrolled in the Nursing Department. Nursing students receive education on the management of primary dysmenorrhea as part of the women’s health and diseases nursing course delivered in the fall semester of the third academic year. Therefore, third- and fourth-year young women were included in the sample to ensure a relatively similar level of knowledge and awareness regarding treatment and coping strategies for primary dysmenorrhea between the intervention and control groups.

**Fig. 1:**
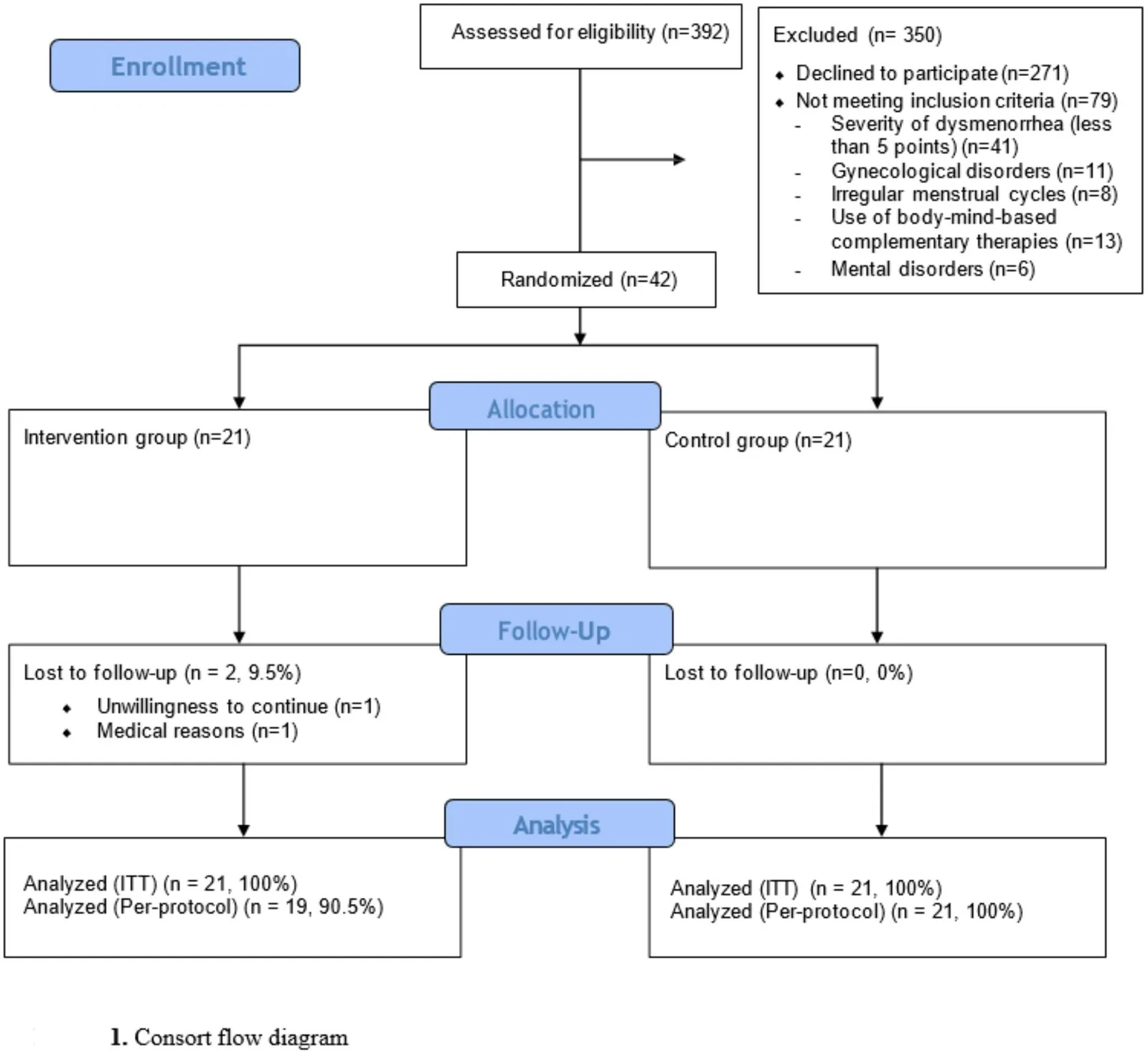
Consort flow diagram

The inclusion criteria were as follows: having primary dysmenorrhea; being nulligravida; having regular menstrual cycles during the previous six months (menstrual cycle length of 21–35 days); not using hormonal contraceptives; reporting a dysmenorrhea pain score of ≥ 5 on the Visual Analog Scale (VAS); not having any diagnosed gynecological, metabolic, cardiovascular, oncological, or psychiatric disorders; not using antidepressant medication; not participating in other mind–body practices; and using a smartphone. Health-related information was obtained based on participants’ self-reports. Eligibility for participation was assessed by the researchers through face-to-face interviews, and 42 young women who met the inclusion criteria were enrolled in the study (Fig. 1).

A post hoc power analysis was performed using G*Power to assess the adequacy of the sample size for the repeated-measures design. Based on the observed effect size for dysmenorrhea pain measured by the VAS (ηp² = 0.109, f = 0.350), with two groups, three measurement points, a total sample size of 42, and an alpha level of 0.05, the statistical power of the study was calculated as 0.99.

### Instruments

Data were collected through the Descriptive Information Form, the Visual Analog Scale (VAS) for dysmenorrhea pain, the Menstrual Symptom Scale, and the Quality of Life Scale Short Form SF-12.

#### Descriptive information form

This form consists of 27 items: age, economic level, body mass index, presence of chronic disease, smoking status, menstrual cycle characteristics and dysmenorrhea, and coping strategies. This form was prepared by researchers in line with the literature [1–4].

#### Visual analog scale (VAS) for dysmenorrhea pain intensity

Dysmenorrhea pain intensity was assessed using the Visual Analog Scale (VAS), a widely used single-item instrument consisting of a 10-cm horizontal line anchored by the descriptors “no pain” (0) and “worst imaginable pain” (10). Participants were instructed to indicate the intensity of their menstrual pain by marking a point on the scale corresponding to their perceived pain level. Higher scores reflected greater pain intensity.

Baseline pain assessment was performed during the first menstrual cycle prior to the initiation of the intervention. Participants in both the intervention and control groups were asked to record their pain intensity once daily during the first three days of menstruation, which represent the period when dysmenorrhea symptoms are typically most pronounced.

Following the baseline assessment (1st menstrual cycle), the intervention group received progressive muscle relaxation exercises, while the control group continued with their usual daily routines without receiving any structured intervention. Pain assessments were repeated during the first three days of menstruation in each of the subsequent two menstrual cycles (2nd menstrual cycle and 3rd menstrual cycle). Participants in the intervention group recorded their pain intensity after practicing the relaxation exercises according to the study protocol, whereas participants in the control group recorded their pain intensity under routine conditions.

This repeated-measurement approach enabled the evaluation of both between-group differences and within-group changes in dysmenorrhea pain intensity over time.

#### Menstruation symptom scale

The Menstruation Symptom Scale (MSS) was developed to evaluate menstrual pain and symptoms [22]. The original version of the scale has 24 items and a five-point Likert scale. The Turkish validity and reliability study of the scale was conducted by Güvenç et al. (2014) [23]. The scale is a five-point Likert scale consisting of 22 items and three sub-dimensions in the Turkish version (Negative Effects/Somatic Complaints, Menstrual Pain Symptoms, and Coping Methods). The first 13 items of the scale are in the ‘negative effects/somatic complaints of pain’ sub-dimension, the 14th-19th items are in the ‘menstrual pain symptoms’ sub-dimension, and the 19th-22nd items are in the ‘coping methods’ sub-dimension. The scale score was calculated by calculating the total mean scores of the items in the scale. An increase in the score obtained from the sub-dimensions of scale indicates an increase in the intensity of menstruation symptoms; a decrease in the score indicates a decrease in intensity. The Cronbach alpha value in this scale’s Turkish validity and reliability study is 0.92. Cronbach’s alpha value of 0,93 was found for this study.

#### Quality of life scale short form SF-12

The Quality of Life Scale Short Form SF-12 was developed by shortening the short SF-36 form, which Ware et al. (1995) developed to assess quality of life [24]. It includes the same sub-dimensions as the SF-36. SF-12 consists of 8 sub-dimensions and 12 items, including physical functioning (2 items), physical role (2 items), body pain (1 item), general health (1 item), energy (1 item), social functioning (1 item), emotional role (2 items) and mental health (2 items). In the scale, sub-dimensions can be grouped and evaluated as physical (PSC-12) and mental component (MSC-12) scores. The PSC-12 score is obtained from the sub-dimensions of general health, physical functioning, physical role, and body pain. In contrast, the MSC-12 score is obtained from social functioning, emotional role, mental health, and energy sub-dimensions. Scores range from 0 to 100, with higher scores representing better health. The Turkish validity and reliability study of the SF-12 scale was conducted by Soylu and Kütük (2021) [25]. In the Turkish validity and reliability study of the scale, Cronbach’s alpha value was 0.73 for PSC-12 and 0.72 for MSC-12. For this study, Cronbach’s alpha values were 0,79 for the PSC-12 and MSC-12.

#### PMRE practice adherence log

To ensure intervention adherence and fidelity, a structured adherence log was developed for participants in the intervention group. The log was designed to document daily engagement with progressive muscle relaxation exercises (PMRE) throughout the intervention period. It included basic participant identification information and the date of initiation of the PMRE practice.

Separate adherence logs were prepared for each of the two menstrual cycles during which the intervention was implemented. Participants were instructed to record their PMRE practice on a daily basis in accordance with the study protocol. These self-reported records were periodically reviewed by the researchers to monitor adherence to the intervention and to support the consistency of exercise implementation.

### Randomization and blinding

Participants who met the inclusion criteria and provided written informed consent were randomly assigned to either the intervention or the control group. The random allocation sequence was generated by an independent researcher who was not involved in participant recruitment, intervention delivery, or outcome assessment, using a computerized random sequence generator (MATLAB R2022a, The MathWorks Inc., USA). Participants were allocated to the intervention and control groups in a 1:1 ratio according to the generated randomization list. Blinding of participants was not feasible due to the nature of the intervention. Participants in the intervention group self-administered progressive muscle relaxation exercises using standardized audio recordings (Voice Records of Relaxation Exercises CD), and outcome measures were obtained through self-report. To minimize potential reporting and analytical bias, all study data were entered into the statistical software (SPSS) by an independent researcher who was not involved in the intervention process. Group allocation was coded as “A” and “B,” and the allocation list was concealed from the statistician until the completion of data analysis.

### Data collection

Data were collected between May and August 2022. All eligible participants were informed about the purpose and procedures of the study, and written informed consent was obtained prior to enrollment. Participants who met the inclusion criteria were first asked to complete the Menstrual Symptom Scale and the Short Form-12 (SF-12) as baseline assessments. Following the baseline (1st menstrual cycle) measurements, participants were randomly assigned to either the intervention group or the control group.

#### Intervention group

Participants in the intervention group attended an introductory session conducted in a designated classroom within the faculty building. During this session, participants were seated comfortably in armchairs and received standardized verbal instruction regarding progressive muscle relaxation exercises (PMRE) for approximately 10 min, followed by a supervised practice session lasting 30 min.

For baseline assessment, participants were instructed to record their dysmenorrhea pain intensity using the Visual Analog Scale (VAS) during the first three days of the first menstrual cycle without performing any intervention. Subsequently, participants were asked to practice PMRE at home before bedtime at least four times per week over the following two menstrual cycles. In addition, they were instructed to perform the exercises during the first three days of menstruation, when dysmenorrhea symptoms were expected to be most intense. Participants recorded their post-exercise pain scores using the VAS during these periods.

To facilitate adherence and follow-up, participants were included in an online communication group and received regular reminders and encouragement from the researchers. Standardized audio recordings (“Voice Records of Relaxation Exercises”) prepared by the Turkish Psychologists Association were transferred to participants’ smartphones to ensure consistency in intervention delivery.

PMRE sessions lasted approximately 30 min and were conducted according to the instructions provided in the audio recordings. The exercises involved sequential tensing and relaxing of major muscle groups, including the face, upper and lower extremities, neck, shoulders, chest, abdomen, hips, and feet, coordinated with controlled breathing and relaxation cues.

Following completion of the two-menstrual cycle intervention period, Post-intervention assessment (3rd menstrual cycle) using the Menstrual Symptom Scale and the Short Form-12 (SF-12) questionnaire were conducted at the end of the third menstrual cycle. Participants were also monitored for potential adverse effects or discomfort during the intervention period, and no intervention-related adverse events were reported.

#### Control group

Participants in the control group received routine care and did not undergo any additional sham or attention-control intervention during the study period. They were instructed to record their dysmenorrhea pain intensity using the Visual Analog Scale (VAS) on each of the first three days of menstruation across three consecutive menstrual cycles. Follow-up assessments using the Menstrual Symptom Scale and the Short Form-12 (SF-12) questionnaire were conducted at the end of the third menstrual cycle.

### Data analysis

Statistical analyses were conducted using IBM SPSS Statistics version 25 (IBM Corp., Armonk, NY, USA). The normality of data distribution was assessed using the Shapiro–Wilk test. Descriptive statistics were calculated for all study variables. Between-group comparisons of baseline characteristics and outcome variables were performed using independent-samples t-tests and one-way analysis of variance (ANOVA), as appropriate. Within-group changes over time were evaluated using paired-samples t-tests. To examine changes in dysmenorrhea intensity across the three measurement points, repeated-measures two-way analysis of variance (group × time) was conducted using the mean Visual Analog Scale (VAS) scores of participants in the intervention and control groups.

An intention-to-treat (ITT) analysis was performed to preserve the benefits of randomization and minimize potential bias related to participant attrition. All randomized participants were included in the analysis according to their original group allocation, regardless of their level of adherence to the intervention. For participants with missing post-intervention data, missing values were handled using the expectation–maximization (EM) method in SPSS. The assumption of missing completely at random (MCAR) was assessed using Little’s test prior to imputation. This approach ensured that all available baseline data were retained in the final analysis.

### Statement of ethics

Ethical approval for the study was obtained from the Gazi University Ethics Committee (Approval No: 16.03.2022-E-77082166-604.01.02-313563). Written institutional permission was also secured from the faculty where the research was conducted. All participants were informed about the purpose and procedures of the study, and written informed consent was obtained prior to participation. To minimize potential power imbalance and coercion, the researchers had no teaching or evaluative role over the participants during the study period. Participation in the study was entirely voluntary, and the participants were not enrolled in any courses conducted by the researchers at the time of data collection.

The study was conducted in accordance with the ethical principles outlined in the Declaration of Helsinki. Following completion of the study, participants in the control group who wished to receive the intervention were provided with the relaxation exercise audio recordings, and their questions regarding the procedure were addressed by the researchers.

## Results

Participant attrition was low in both groups. In the intervention group, 2 participants (9.5%) discontinued the study due to unwillingness to continue (*n* = 1) and medical reasons (*n* = 1), while no loss to follow-up occurred in the control group. Overall, the low attrition rate suggests good participant retention throughout the study.

### Sociodemographic and clinical characteristics of participants

No statistically significant differences were observed between the intervention and control groups with respect to age, body mass index (BMI), income level, smoking status, age at onset of dysmenorrhea, presence of chronic disease, and use of analgesics for dysmenorrhea (*p* > .05). The baseline characteristics of the participants are presented in Table 1.

**Table 1:** Characteristics of participants with primary dysmenorrhea (*n* = 42)

Characteristics	Intervention Group (*n* = 21)	Control Group (*n* = 21)	Test	*p*
Age, years	21.76 (0.99)	21.61 (1.24)	t	0.393
BMI (kg/m²)	21.44 (3.57)	21.61 (3.36)	t	0.441
BMI category, n (%)			χ²	0.595
Underweight	2 (9.5)	4 (19.0)		
Normal	18 (85.7)	14 (66.7)		
Overweight	–	2 (9.5)		
Obese	1 (4.8)	1 (4.8)		
Economic status, n (%)			χ²	0.500
Low	2 (9.5)	3 (14.2)		
Middle	18 (85.7)	17 (81.0)		
High	1 (4.8)	1 (4.8)		
Smoking status, n (%)			χ²	0.172
Yes	1 (4.8)	4 (19.0)		
No	20 (95.2)	17 (81.0)		
Onset of dysmenorrhea, n (%)			χ²	0.135
First day of menstruation	9 (42.9)	6 (28.6)		
A few hours before menstruation	7 (33.3)	6 (28.6)		
A few days before menstruation	5 (23.8)	9 (42.8)		
Chronic disease, n (%)			χ²	0.116
Yes	3 (14.3)	–		
No	18 (85.7)	21 (100.0)		
Use of analgesics, n (%)			χ²	0.500
Yes	14 (66.7)	13 (61.9)		
No	7 (33.3)	8 (38.1)		

Values are presented as mean (SD) for continuous variables and n (%) for categorical variables. Independent-samples t-test and chi-square test were used as appropriate. BMI: body mass index

### Pain intensity, menstruation symptom intensity, and quality of life levels of the participants

The analysis showed no significant between-group differences at baseline (1st menstrual cycle) for any of the three outcome assessments (*p* > .05). However, at both the 2nd and 3rd menstrual cycle assessments, the mean scores were significantly lower in the intervention group compared with the control group across all three measures (*p* < .05).

Within-group comparisons indicated no significant changes over time in the control group (*p* > .05), whereas significant reductions were observed in the intervention group across all assessments (*p* < .05). Post hoc analyses demonstrated that these differences were primarily attributable to significantly higher baseline scores compared with both the 2nd and 3rd cycle measurements (baseline > 2nd menstrual cycle and baseline > 3rd menstrual cycle; *p* < .01).

**Fig. 2:**
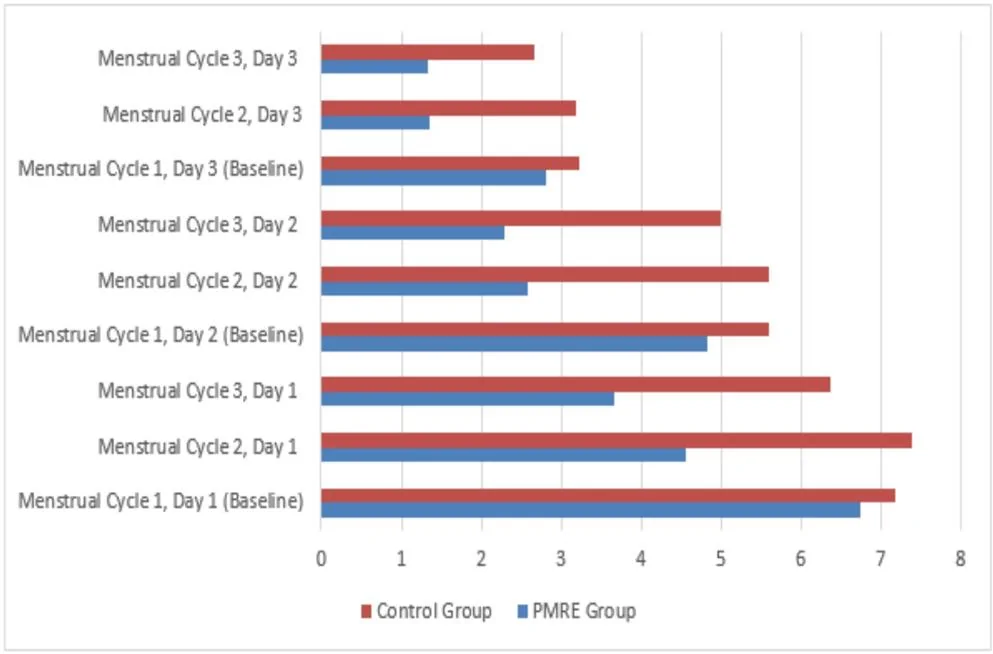
Changes in dysmenorrhea pain intensity measure by VAS across menstrual cycles in the intervention and control groups

Repeated-measures analyses further revealed significant main effects of group in the first and second assessments (*p* < .01). In addition, significant time effects and group × time interactions were identified for all three outcome measures (*p* < .05), indicating that dysmenorrhea intensity decreased over time in the intervention group compared with the control group (Table 2, Figure 2).

**Table 2:** Comparison of Dysmenorrhea Intensity Levels Between the Intervention and Control Groups (*n* = 42)

Measurement	Time Point	Intervention Group (*n* = 21) Mean ± SD	Control Group (*n* = 21) Mean ± SD	Test	*p*	ηp²
First day	Baseline (Cycle 1)	6.76 ± 1.87	7.19 ± 1.94	F = 0.538	0.468	0.013
	Cycle 2	4.57 ± 2.01	7.38 ± 1.96	F = 21.024	< 0.001	0.345
	Cycle 3	3.67 ± 2.20	6.38 ± 2.56	F = 13.562	0.001	0.253
	Within-group (Intervention)	–	–	F = 30.447	< 0.001	0.610
	Within-group (Control)	–	–	F = 1.703	0.195	0.080
	Group effect	–	–	F = 14.560	< 0.001	0.267
	Time effect	–	–	F = 16.834	< 0.001	0.296
	Group × Time	–	–	F = 8.027	0.001	0.167
Second day	Baseline (Cycle 1)	4.83 ± 2.25	5.62 ± 2.54	F = 1.132	0.294	0.028
	Cycle 2	2.58 ± 1.87	5.62 ± 2.40	F = 20.990	< 0.001	0.344
	Cycle 3	2.30 ± 2.06	5.00 ± 2.43	F = 15.116	< 0.001	0.274
	Within-group (Intervention)	–	–	F = 21.417	< 0.001	0.523
	Within-group (Control)	–	–	F = 1.474	0.241	0.070
	Group effect	–	–	F = 12.755	0.001	0.242
	Time effect	–	–	F = 14.722	< 0.001	0.269
	Group × Time	–	–	F = 8.232	0.001	0.171
Third day	Baseline (Cycle 1)	2.81 ± 2.60	3.24 ± 2.64	F = 0.277	0.601	0.007
	Cycle 2	1.36 ± 1.67	3.19 ± 2.71	F = 6.944	0.012	0.148
	Cycle 3	1.33 ± 1.71	2.67 ± 2.18	F = 4.870	0.033	0.109
	Within-group (Intervention)	–	–	F = 8.033	0.001	0.292
	Within-group (Control)	–	–	F = 1.171	0.321	0.057
	Group effect	–	–	F = 3.621	0.064	0.083
	Time effect	–	–	F = 7.122	0.001	0.151
	Group × Time	–	–	F = 3.209	0.046	0.074

Values are presented as mean ± SD. Repeated-measures two-way ANOVA (group × time) was used. ηp²: partial eta squared

At baseline (1st menstrual cycle), there was no significant difference in the mean Menstrual Symptom Scale (MSS) scores between the PMRE and control groups (*p* = .177). However, at the post-intervention assessment (3rd menstrual cycle), the mean MSS score was significantly lower in the intervention group compared with the control group (*p* < .001). The magnitude of the intervention effect was large (Cohen’s d = 1.461), whereas the effect size observed in the control group was small (d = 0.304) (Table 3). Within-group comparisons showed a significant reduction in mean MSS scores in the intervention group from pretest to posttest (*p* = .009). In contrast, no significant change was observed in the control group (*p* > .05) (Table 3).

**Table 3:** Comparison of Mean Menstruation Symptom Scale (MSS) and Quality of Life Short Form (SF-12) Scores Between the Intervention and Control Groups (*n* = 42)

Variables	Time point	Intervention Group (*n* = 21) Mean (SD)	Control Group (*n* = 21) Mean (SD)	Test	*p*	Effect size (d)
MSS	Baseline (Cycle 1)	72.63 (22.54)	78.14 (12.07)	t = 1.374	0.177	0.304
	Post (Cycle 3)	56.42 (15.87)	79.57 (15.80)	t = 4.616	< 0.001	1.461
	Within-group (Intervention)	–	–	t = 2.918	0.009	0.831
	Within-group (Control)	–	–	t=-0.560	0.517	0.101
MSC-12	Baseline (Cycle 1)	43.61 (10.38)	37.10 (12.41)	t=-1.844	0.073	0.569
	Post (Cycle 3)	49.24 (6.11)	38.19 (12.21)	t=-3.706	0.001	1.144
	Within-group (Intervention)	–	–	t=-2.619	0.016	0.661
	Within-group (Control)	–	–	t=-0.594	0.559	0.088
PSC-12	Baseline (Cycle 1)	48.17 (7.63)	45.97 (6.60)	t=-1.000	0.323	0.308
	Post (Cycle 3)	49.70 (5.35)	47.71 (7.22)	t=-1.011	0.318	0.313
	Within-group (Intervention)	–	–	t=-1.231	0.233	0.232
	Within-group (Control)	–	–	t=-0.892	0.383	0.251

Values are presented as mean (SD). Independent-samples t-test was used for between-group comparisons, and paired-samples t-test was used for within-group comparisons. MSS: Menstrual Symptom Scale; MSC-12: Mental Component of SF-12; PSC-12: Physical Component of SF-12

Quality of life was evaluated using the mental (MSC-12) and physical (PSC-12) component scores of the Short Form-12. At baseline (1st menstrual cycle), there were no significant differences between the PMRE and control groups in either MSC-12 or PSC-12 mean scores (*p* > .05).

At the post-intervention assessment (3rd menstrual cycle), the intervention group demonstrated significantly higher mean MSC-12 scores compared with the control group (*p* = .001). In contrast, no significant between-group differences were observed in PSC-12 scores (*p* = .323 and *p* = .318) (Table 3).

## Discussion

This study investigated the effects of PMRE on pain, menstrual symptoms, and quality of life. The results indicated that PMRE alleviated menstrual symptoms and pain intensity, and increased mental health quality of life, although it did not affect physical health quality of life.

Dysmenorrhea is an important factor that negatively affects the quality of life of young women [26–28]. In this study, it was found that the intensity of dysmenorrhea in the first three days of the two cycles following PMRE was lower in those who performed PMRE than in those who did not perform PMRE. Also, time and group-time interactions were significant for all assessments of the intensity of dysmenorrhea. Similarly, Celenay et al. (2024) found that menstrual pain intensity was lower in those who practiced PMRE than in those who did not practice PMRE [21]. In another similar study, women had less dysmenorrhea pain in the two cycles following PMRE [20]. In addition, a study by İbrahim et al. (2023) found that relaxation exercises were more effective in coping with dysmenorrhea than warm compresses [29]. Pawestri et al. (2023) compared the effect of PMRE and endorphin massage on menstrual pain in adolescents and found that PMRE was more effective than endorphin massage in controlling menstrual pain [30]. This finding of the study is similar to those reported in the literature. PMRE can make it easier to deal with pain by increasing the effectiveness of the parasympathetic nervous system, the emission of β-endorphins, which are natural pain relievers, and the decrease of muscle tension and pain perception [14, 31–33]. In our study, the decrease in the intensity of dysmenorrhea might have resulted from the positive physiological and psychological processes provided by PMRE.

In this study, it was found that PMRE decreased the intensity of menstrual symptoms in women with dysmenorrhea. There are some research findings in the literature that support our study results. In the study of Çitil and Canbay (2024), it was reported that the intensity of premenstrual syndrome decreased in those who performed PCI compared to those who did not [34]. Similar to our results, another study determined that PKGE administration alleviated menstrual symptom intensity in women [21]. In the study of Yıldız and Acaroğlu (2022), it was determined that massage therapy given to women with dysmenorrhea, together with progressive muscle relaxation exercises, reduced physiological and psychological negative menstrual symptoms [17]. PMRE plays a role in manipulating autonomic nervous system responses, activating and/or increasing opioid production, and supporting optimal immune function [14]. PMRE, one of the body-mind medicine techniques, may have a positive effect on menstrual symptoms by positively affecting the physiological and psychological processes of women with dysmenorrhea [14]. In our study, the reduction in the intensity of dysmenorrhea by PMRE may have had a positive effect on the reduction of menstrual symptom intensity.

Non-pharmacological methods, including mind and body techniques, facilitate coping with pain, reduce pain perception, and promote relaxation [32]. In this study, it was found that PMRE improved the mental quality of life of young women with dysmenorrhea; however, it did not affect the physical quality of life. In the study of Celenay et al. (2024), it was determined that PMRE intervention improved the quality of life of women [21]. In a study conducted by Ganesh et al. (2017), two different relaxation techniques were compared, and it was reported that the progressive relaxation technique was more effective in improving quality of life [35]. On the other hand, Ibrahim et al. (2023) compared warm compress and PMRE interventions, finding that women in the warm compress group showed higher quality of life in general health and emotional well-being compared to the PMRE group [29]. While our findings are similar to some of the non-pharmacological intervention studies in the literature, they differ from others. Pain perception is an important determinant of physical and mental quality of life [36]. In this study, PMRE was applied to relax the body and mind, and the intensity of dysmenorrhea decreased. This might have contributed to improving women’s mental quality of life.

However, the lack of a significant change in the physical component of quality of life may be explained by several factors. First, the participants were young individuals with relatively good baseline physical functioning, which may have limited the potential for measurable improvement [28]. Second, changes in physical health-related quality of life outcomes may require longer intervention periods or more comprehensive lifestyle-based approaches than those applied in the present study [5]. Third, mind–body interventions such as PMRE are more likely to exert early effects on psychological well-being, stress regulation, and pain perception than on broader physical health status [37]. In addition, the absence of a significant improvement in physical quality of life may also be related to a possible ceiling effect, given that participants demonstrated relatively high baseline physical health scores, limiting the potential for further measurable gains. Furthermore, the use of different measurement tools across studies and variations in intervention duration may also contribute to inconsistencies in findings.

Participant attrition in this study was minimal and occurred due to unwillingness to continue (*n* = 1) and medical reasons (*n* = 1). Such losses are commonly reported in intervention studies and may be related to individual motivation, perceived burden of participation, or unforeseen health conditions. Although the number of dropouts was low, these factors may have influenced adherence to the intervention. However, the use of an intention-to-treat analysis helped to minimize potential bias related to attrition and preserve the validity of the findings.

## Conclusion

In conclusion, progressive muscle relaxation exercises were associated with reductions in pain intensity and menstrual symptoms, as well as improvements in the mental component of quality of life among young women with primary dysmenorrhea. These findings suggest that PMRE may represent a feasible and supportive non-pharmacological strategy for symptom management in this population. In addition, the absence of a significant improvement in physical quality of life may be related to the relatively short duration of the intervention, the participants’ generally good baseline physical health status, and the multidimensional nature of physical health outcomes.

Encouraging women to actively engage in healthy lifestyle behaviors and structured relaxation-based exercises may contribute to improved symptom control and psychological well-being. With appropriate counseling and guidance, PMRE may help reduce menstrual symptom intensity and enhance mental quality of life. Considering its practicality, low cost, and ease of implementation, PMRE may be considered a complementary approach in clinical practice. Further research including larger and more diverse populations and longer follow-up periods is recommended to strengthen the evidence regarding the effectiveness of PMRE in primary dysmenorrhea management.

### Limitations & strengthens

 One of limitations of this study is that the sample consisted exclusively of nursing students. As these students receive education related to health promotion and dysmenorrhea management, they may be more knowledgeable about symptom control strategies and more receptive to behavioral interventions. Therefore, the findings of this study should be interpreted with caution in terms of generalizability. Future research including participants from different educational backgrounds and community settings is recommended. Another limitation of the study, participant attrition, although minimal, should be considered a limitation, as withdrawal due to personal or medical reasons may introduce potential bias and limit the generalizability of the findings.

## Data Availability

The data supporting this study’s findings are available upon reasonable request.

## Declarations

### Peer-review

Externally peer-reviewed.
